# Risk factors for oral mucositis during chemotherapy treatment for solid tumors: a retrospective STROBE-guided study

**DOI:** 10.4317/medoral.25253

**Published:** 2022-06-19

**Authors:** Joyce Ohana de Lima Martins, Marcela Maria Fontes Borges, Cássia Emanuella Nóbrega Malta, Anna Clara Aragão Matos Carlos, André Alves Crispim, José Fernando Bastos de Moura, Isabelle Joyce Silva Fernandes-Lima, Paulo Goberlânio de Barros Silva

**Affiliations:** 1 Dentistry Graduate Program, Federal University of Ceará; 2 https://orcid.org/0000-0001-9185-9491. Dentistry Graduate Program, Federal University of Ceará; 3 Graduate Program in Dental Sciences, Unichristus; 4 Post-Graduate Program in Oncology, Rodolfo Teófilo College/Ceará Cancer Institute

## Abstract

**Background:**

This study retrospectively analyzed the risk factors for transchemotherapy oral mucositis (OM).

**Material and Methods:**

Before each chemotherapy cycle, patients were routinely evaluated for the presence/severity of OM based on the Common Terminology Criteria for Adverse Events (CTCAE) v5.0 scale for adverse effects and graded as follows: However, specific conditions such as mucositis are graded on a five-point scale: 0, absence of mucositis, grade 1 (Asymptomatic or mild), 2 (Presence of pain and moderate ulceration, without interference with food intake), 3 (severe pain with interference with food intake) or 4 (Life-threatening with the need for urgent intervention). Information from 2 years of evaluations was collected and patient medical records were reviewed to obtain data on chemotherapy cycle, sex, age, body mass index, body surface area, primary tumor, chemotherapy protocol, and history of head and neck radiotherapy. The X² test and multinomial logistic regression were used for statistical analysis (SPSS 20.0, *p*<0.05).

**Results:**

Among 19,000 total evaluations of 3,529 patients during 5.32±4.7 chemotherapy cycles (CT) the prevalence of OM was 6.3% (n=1,195). Chemotherapy duration (*p*<0.001), female sex (*p*=0.001), adjuvant intention (*p*=0.008) and the use of carboplatin (*p*=0.001), cisplatin (*p*=0.029), docetaxel (*p*<0.001) and bevacizumab (*p*=0.026) independently increased the risk of mucositis. In head and neck tumors, 2018 year (*p*=0.017), chemotherapy duration (*p*=0.018), BMI>30 (*p*=0.008), radiotherapy (*p*=0.037) and use of carboplatin (*p*=0.046) and cyclophosphamide (*p*=0.010) increased this prevalence.

**Conclusions:**

Cycles of chemotherapy, sex, cytotoxicity drugs, bevacizumab and head and neck radiotherapy increase the risk of OM in solid tumors.

** Key words:**Mucositis, stomatitis, antineoplastic agentes, neoplasms, antineoplastic combined chemotherapy protocols.

## Introduction

The increasing effectiveness of chemotherapy protocols for cancer treatment has been increasing the use of drug combinations with surgery and radiotherapy to treat solid tumors (1). Chemotherapy for solid tumors is usually associated with combining one or more antineoplastic drugs that have different pathways to interfere in the division of malignant cells (2-4). The efficacy of treatments increases with different dosages and with different combinations of chemotherapeutics. However, along with the increased clinical benefit in controlling tumor growth, these combinations increase the incidence of several adverse effects (5).

The adverse effects of chemotherapy are systemic but transient. They are associated with organs and tissues with a high capacity of cell replication and, depending on the severity, may require the interruption of treatment (6). The emergence of oral lesions is common in antineoplastic treatment because the tissues of the oral cavity have a high metabolic capacity, becoming sensitive to cytotoxic damage from treatment (7), among them oral mucositis is the main dose- and treatment-limiting effect in the oral cavity (8).

Chemotherapy-induced oral mucositis is an adverse effect of chemotherapy, radiotherapy (head and neck), or a combination of both, which can affect 20% to 40% of patients receiving chemotherapy for solid tumors and up to 80% of patients being treated for myeloproliferative disorders. Its incidence significantly increases when there is a combination with the head and neck radiotherapy (5,9), and its pathogenesis occurs both by direct tissue damage of the antineoplastic in oral cavity epithelia and by the formation of reactive oxygen species arising from this damage (10).

Clinically, oral mucositis presents as ulcerative lesions of erythematous nature, and its severity can vary from grade 1 (Asymptomatic or mild) to Grade 4 (Life-threatening with the need for urgent intervention) (CTCAE v5.0) (11). Severe oral mucositis requires immediate treatment, requiring dose reduction, or interruption of chemotherapy, affecting the patient's prognosis and quality of life and generating a strong economic impact associated with hospitalization support (6).

Some risk factors for oral mucositis have been described, such as age and gender (12), time of diagnosis, previous history of (13), and some specific drugs are more strongly associated with oral mucositis than others (14). Additionally, numerous ways to decrease the incidence of oral mucositis (15), such as low-intensity laser therapy (16), have been tested. However, all proposed therapies present a cost or require specialized human resources for their realization (15), making risk factor recognition strategies indispensable for directing targeted and individualized therapeutic/prophylactic conduct.

Thus, given the increase in the use of chemotherapy for the treatment of solid tumors and the importance that the recognition of risk factors for oral mucositis has for the implementation of prophylactic measures, this study aims to evaluate the incidence of oral mucositis during chemotherapy for solid tumors and investigate its risk factors.

## Material and Methods

- Study design and scenario

This observational, retrospective, cross-sectional, and quantitative study was guided by the STROBE initiative, an international guideline for reporting observational studies (17). This study was performed using data on adverse effects in the oral cavity and mucositis during chemotherapy treatment that was collected from the electronic patient record system at Haroldo Juaçaba Hospital/Ceará Cancer Institute (HHJ/ICC) over two years (January 1, 2018, to March 12, 2019).

- Inclusion and exclusion criteria

The inclusion criteria were patients evaluated by a multi-professional team of the HHJ/ICC for side effects during chemotherapy performed between January 1, 2017, and December 31, 2018. The members of the multi-professional team of the HHJ/ICC chemotherapy outpatient clinic routinely evaluated mucositis in patients before each chemotherapy session, recorded the severity scores using the toxicity scales tool, and classified them based on their degree of severity.

The exclusion criteria were patients undergoing treatment for myeloproliferative disorders, occult or metastatic disease with an unknown primary site, as well as those with medical records lacking clinical information required for the assessment of risk factors. Repeated patients (>1 evaluation) were also excluded.

- Collection of socio-demographic and clinical data

With the number of services provided by the Tasy system's toxicity scale tool, a manual search of each service's records was performed to retrieve the clinical and pathological data of interest. Patients appearing more than once were ordered by their date of care to identify the number of chemotherapy cycles.

During the manual collection of information based on the number of care visits, the patients’ medical records were collected, as well as age, sex, weight on the day of care, height, chemotherapy purpose (neoadjuvant, adjuvant, or palliative), clinical stage, chemotherapy protocol, and primary tumor location. Additionally, the tumor-node-metastasis (TNM)-2016 grading system (18) was used to classify the stage of the solid tumors. Information on previous/concomitant head and neck radiotherapy was obtained from patients with head and neck tumors. All data were recorded using a Microsoft Excel spreadsheet.

- Adverse effects analysis tool

The toxicity scale tool used was the Common Terminology Criteria for Adverse Events (CTCAE). Formerly called the Common Toxicity Criteria (CTC or NCI (National Cancer Institute)-CTC), this tool contains a set of criteria for the standardized classification of adverse effects of drugs used in cancer therapy. The CTCAE system is a product of the US National Cancer Institute (NCI) and has been widely used in many clinical trials, extending beyond oncology and encoding their observations based on the CTCAE system (11).

The CTCAE system toxicity scale includes the following adverse effects: mucositis, vomiting index, diarrhea, nausea, constipation, anorexia, dysgeusia, alopecia, hand and foot syndrome, fatigue, insomnia, and dysuria. All patients were classified according to toxicity scores suggested by the Common Terminology Criteria for Adverse Events (CTCAE) v5.0 scale for adverse effects. It uses a range of grades from 1 to 5. However, specific conditions such as mucositis are graded on a five-point scale: 0, absence of mucositis, grade 1 (Asymptomatic or mild), 2 (Presence of pain and moderate ulceration, without interference with food intake), 3 (severe pain with interference with food intake) or 4 (Life-threatening with the need for urgent intervention) (11).

After each medical consultation performed immediately before chemotherapy, the multi-professional team assigned the following toxicity grades for mucositis, which were registered in the toxicity scale tool and exported to a standard Microsoft Excel spreadsheet containing the number and date of attendance and the degree of severity of the adverse effect. Patients with >1 evaluation were excluded.

- Statistical approach

The data were exported to IBM SPSS Statistics for Windows, Version 20.0, to perform the statistical analyses and obtain 95% confidence intervals.

The prevalence of oral mucositis grades was expressed as an absolute frequency and percentage compared to the risk factors using Fisher's exact or Pearson's chi-square tests. Variables with *p*<0.200 were then included in a multinomial logistic regression model (multivariate analysis).

## Results

- Clinical characteristics and clinical risk factors for oral mucositis during chemotherapy treatment of patients with solid tumors

A total of 19,839 mucositis evaluations were surveyed in this study, of which 395 were excluded because they were tumors of unknown primary origin, 319 because they were evaluations of patients undergoing treatment for leukemia/lymphomas, and 125 were on treatment with hormone inhibitors. Thus, 19,000 patient evaluations were included from 3,529 patients.

Patients underwent a mean of 5.32±4.77 cycles of chemotherapy, with a median of 4 cycles, ranging from 1 to 41 cycles of chemotherapy. The prevalence of mucositis was 6.3% (n=1,195), of which 975 assessments had grade 1 mucositis, 109 had grade 2 mucositis, and 109 patients had grade 3 mucositis. One patient reached two assessment times with grade 4 mucositis; a woman with breast cancer who had undergone five cycles of chemotherapy with trastuzumab, in which she scored 0, and four cycles with docetaxel plus trastuzumab, in which she scored 1 in the sixth cycle, score 3 in the seventh cycle, and score 4 in the eighth and ninth cycles.

Most patients were included in 2019 (56.7%) and initially seen in the morning shift (89.7%). Most patients had between 6 and 10 cycles of chemotherapy (21.7%), were female (74.1%), aged 56-65 years (27.7%), and BMI between 18.2 and 25.0 (40.0%). From the year 2018 to 2019, there was a significant decrease in the incidence of oral mucositis (*p*<0.001), patients seen in the afternoon shift had a higher incidence of this outcome (*p*<0.001), and the number of cycles of chemotherapy was directly associated with increased incidence of mucositis (*p*<0.001) (Table 1).

Women (*p*<0.001), patients between 45-55 years (*p*=0.005), and high BMI (*p*=0.033) were at higher risk for mucositis. In multivariate analysis, the number of cycles of chemotherapy was the factor that most increased the incidence of mucositis, with patients with more than ten cycles of chemotherapy having an increased risk by 103.40 (CI95% = 38.18-280.08) times independent of the other variables. The year 2018, female gender, and age between 45-55 years (Table 1).

- Tumor profile and tumor staging as a clinical risk factor for oral mucositis during chemotherapy treatment of patients with solid tumors

Regarding the clinical characteristics of the tumor, tumors of the head and neck (*p*<0.001), breast (*p*<0.001), sarcomas (*p*=0.014), liver (*p*<0.001) were directly associated with oral mucositis, while colorectal tumors (*p*<0.001), in the stomach (*p*=0.018), cervix (*p*<0.001), esophagus (*p*=0.014), ovary (*p*<0.001) and pancreas (*p*=0.018) were inversely associated with the presence of oral mucositis. However, the primary tumor location did not influence the risk of oral mucositis in the multivariate analysis (Table 1).

Stage 4 (50.2%), T3 (35.1%), N1 (40.8%) and M0 (70.8%) tumors had the highest prevalence in this sample. The risk of mucositis was significantly increased in stage 1 tumors (*p*<0.001), with T4 size (*p*=0.006) and absence of distant metastasis (*p*<0.001). In multivariate analysis stage 4 tumors had a 2.83 (CI95% = 1.08-7.45) times higher risk of developing oral mucositis and T4 and M0 tumors a 0.21 (CI95% = 0.08-0.63) and 0.38 (CI95% = 0.16-0.88) times lower risk (Table 1).

**Table 1:**
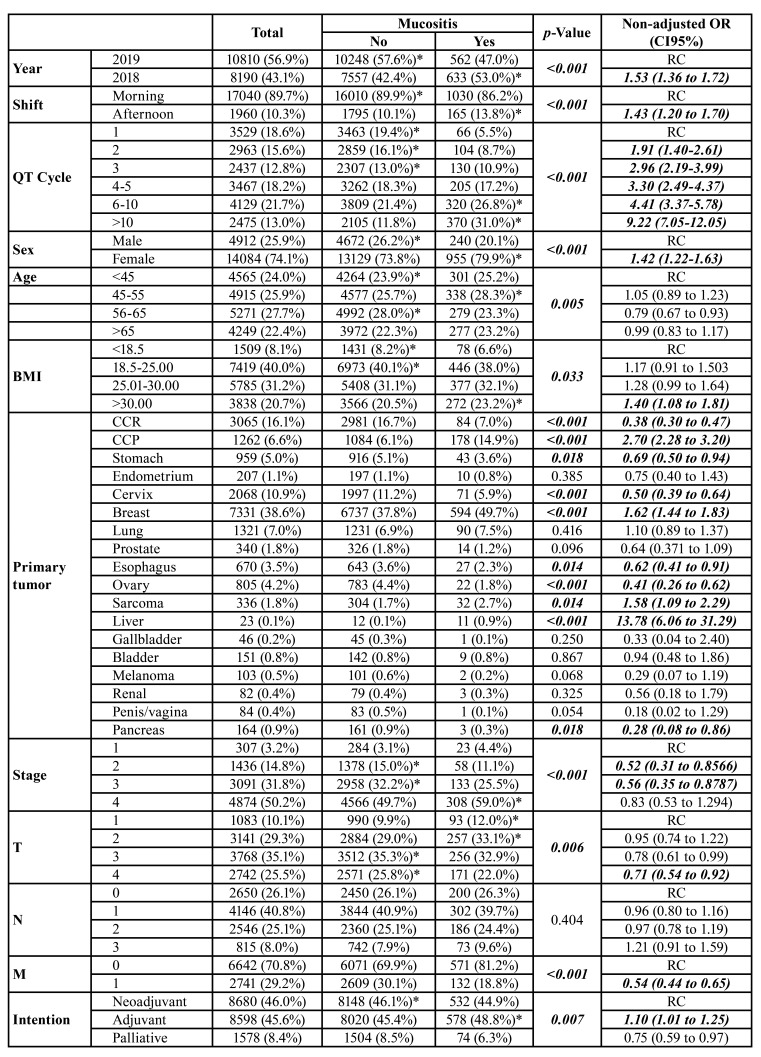
Influence of clinical profile and number of chemotherapy cycles on the prevalence of oral mucositis in patients undergoing chemotherapy treatment for solid tumors.

**Table 1 cont.**
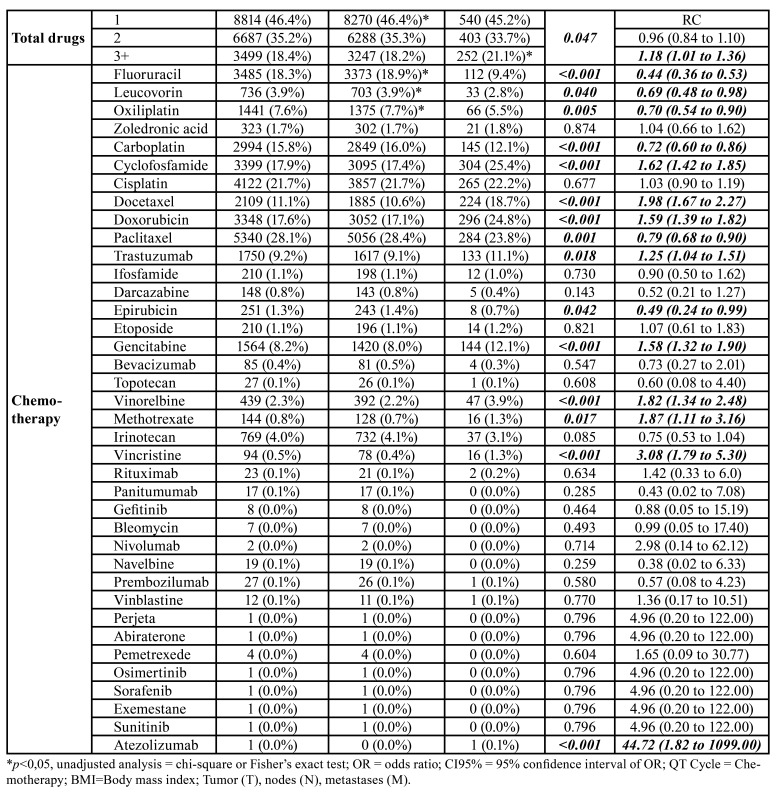
Influence of clinical profile and number of chemotherapy cycles on the prevalence of oral mucositis in patients undergoing chemotherapy treatment for solid tumors.

- Characteristics of chemotherapy for solid tumors that influence the prevalence of oral mucositis

Among the chemotherapy regimens, neoadjuvant (46.0%) followed by adjuvant (45.6%) were the most prevalent intentions. Only 8.4% of patients had palliative treatment. Most patients took monotherapy (46.4%) or combination therapy with two (35.2%), three (16.8%), or four (1.2%) drugs. Only 48 patients did a five-drug combination, 16 patients did a six-drug combination, and nine did seven-drug regimens. The most commonly used drug was paclitaxel (28.1%), followed by cisplatin (21.7%) (Table 1).

Adjuvant treatments had a higher prevalence of oral mucositis (0.007), as did patients who had three or more drugs in their chemotherapy regimen. Cyclophosphamide (*p*<0.001 docetaxel (*p*<0.001)< doxorubicin (*p*<0.001), trastuzumab (*p*=0.018) and Atezolizumab (*p*<0.001) increased the prevalence of mucositis, whereas fluoruracil (*p*<0.001), leucovorin (*p*=0.040), oxaliplatin (*p*=0.005), carboplatin (*p*<0.001), paclitaxel (*p*=0.001), epirubicin (*p*=0.042) were inversely associated with this adverse effect (Table 1).

In multivariate analysis, more than six cycles of chemotherapy and gender were the clinical variables that increased the prevalence of oral mucositis by 9.14 (95% CI = 5.69-14.69) and 3.40 (95% CI = 1.53-7.58) times (Table 2). The presence of distant metastases reduced this prevalence by 0.46 (CI95% = 0.24-0.87) times and adjuvant treatments had an increased prevalence by 1.80 (CI95% = 1.16-2.77) times. Leucovorin was inversely associated with oral mucositis (OR = 0.01, CI95% = 0.00-0.08) and carboplatin (OR = 11.06, CI95% = 2.79-43.87), cisplatin (OR = 2.81, CI95% = 1.11-7.12), docetaxel (OR = 4.21, CI95% = 2.23-7.94) and bevacizumab (OR = 8.25, CI95% = 1.28-53.05) significantly increased the prevalence of mucositis (Table 2).

**Table 2:**
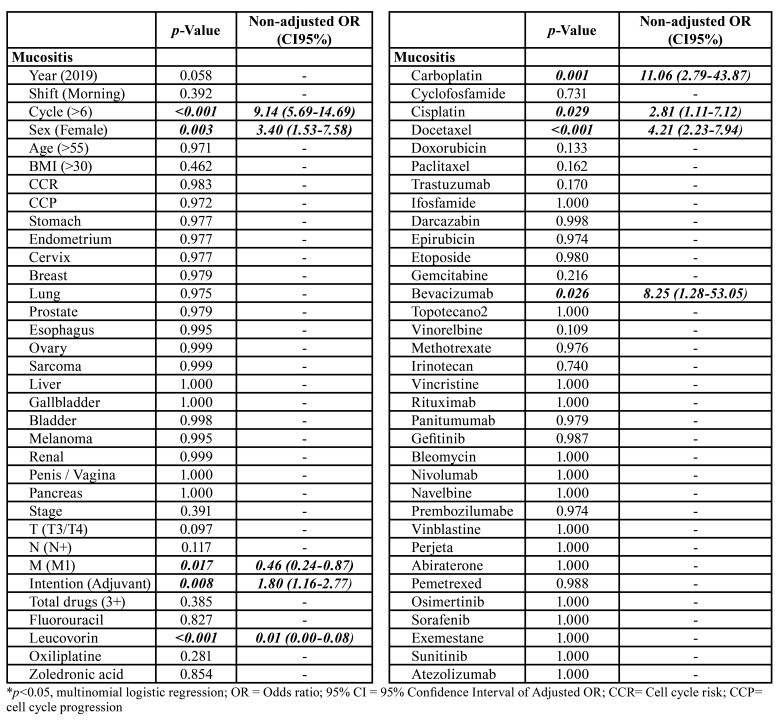
Multivariate analysis of risk factors for oral mucositis in patients undergoing chemotherapy treatment for solid tumors.

- Risk factors for oral mucositis in patients with solid head and neck tumors 

Among the 1,262 patients with head and neck tumors, the majority were seen in the year 2018 (62.4%), in the morning shifts (91.1%), received one or two cycles of chemotherapy (44.1%), were male (72.2%), aged 56-65 years (33.5%) and normal-weight (56.6%). The most prevalent stage was stage IV (78.1%), T4 (45.5%), N2 (36.2%), M0 (80.0%). Neoadjuvant treatment intention was more prevalent (45.5%) than the others, and most patients had chemotherapy with only one drug (57.0%). Head and neck RT was used in most patients (92.9%), and cisplatin (50.4%), followed by carboplatin (36.1%) and paclitaxel (32.3%), were the most commonly used chemotherapy drugs (Table 3).

In patients with head and neck tumors the year 2018 (*p*=0.019), >3 cycles of chemotherapy (*p*<0.001), female gender (*p*<0.001), age (*p*<0.001), and high BMI (*p*=0.021) were directly associated with oral mucositis. T3/T4 (*p*=0.028), N3 (*p*=0.001) tumors also showed an increased prevalence of oral mucositis, and the presence of distant metastases (*p*=0.001) and palliative treatment (*p*<0.001) reduced this prevalence. Monotherapies showed a higher prevalence of mucositis than therapies involving two or more chemotherapies (*p*=0.007), head and neck RT increased the prevalence of oral mucositis (*p*=0.037) and oxaliplatin (*p*=0.006), docetaxel (*p*<0.001), gemcitabine (*p*<0.001), irinotecan (*p*<0.001) were the chemotherapeutics directly associated with oral mucositis, whereas the use of paclitaxel (*p*<0.001), vinorelbine (*p*=0.030) were inversely associated with this adverse effect (Table 3).

In multivariate analysis, 2019 showed a 0.02-fold (0.00-0.52) reduction in the prevalence of oral mucositis and number of cycles of chemotherapy (OR = 303.93, CI95% = 2.69-34385.65), BMI>30 (OR = 481.95, CI95% = 4.88-47599.59) and head and neck RT (OR = 52.25, CI95% = 1.17-4,066.70) significantly increased this prevalence. Carboplatin (OR = 227.33, CI95% = 1.10-47071.54) and Cyclophosphamide (OR = 531.77, CI95% = 4.52-62575.31) were the drugs most strongly associated with this adverse effect (Table 4).

**Table 3:**
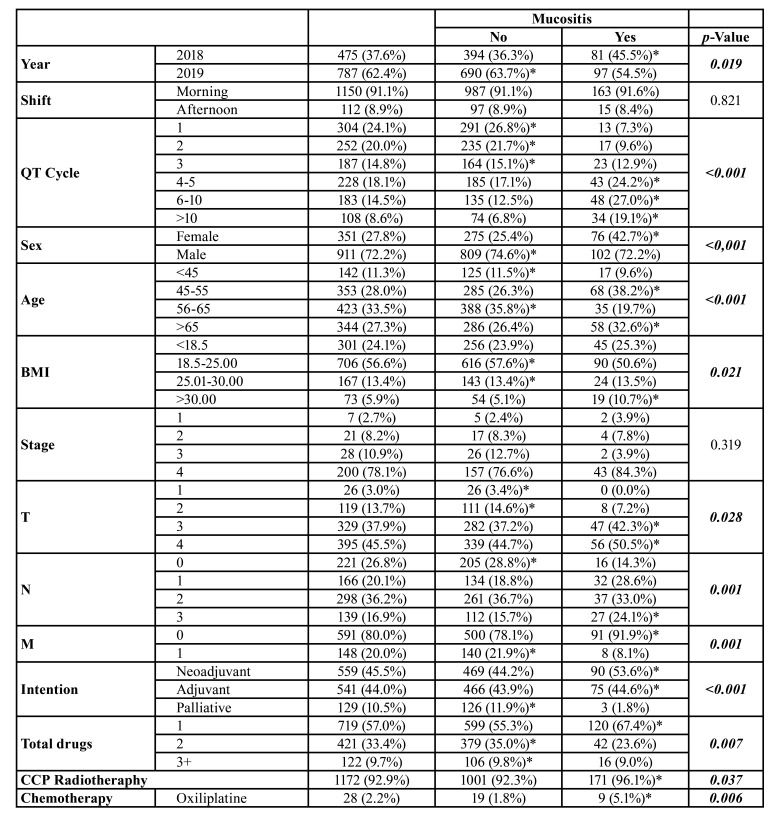
Influence of clinical profile and number of chemotherapy cycles on the prevalence of oral mucositis in patients undergoing chemotherapy treatment for head and neck tumors.

**Table 3 cont.**
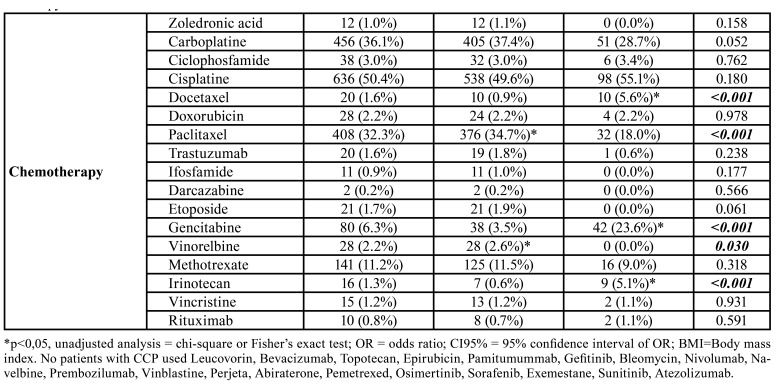
Influence of clinical profile and number of chemotherapy cycles on the prevalence of oral mucositis in patients undergoing chemotherapy treatment for head and neck tumors.

**Table 4:**
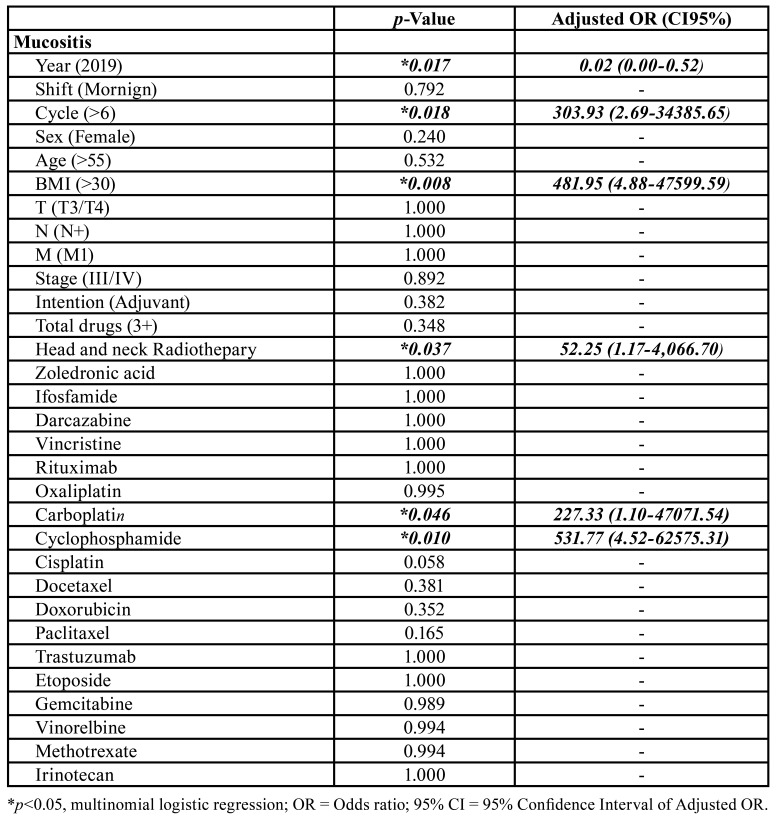
Multivariate analysis of risk factors for oral mucositis in patients undergoing chemotherapy treatment for head and neck tumors.

## Discussion

Few studies comprehensively evaluate chemotherapy treatments for solid tumors and their relation with OM incidence, and its development is underestimated. Thus, this is one of the few studies that comprehensively assess the incidence of oral mucositis and the risk factors for its development during chemotherapy of solid tumors.

Based on 19,000 assessments surveyed, 6.3% (n=1,195) showed OM. This diverges from previous studies, which had an incidence of OM of 40 -81%. (13,19,20). These differences can be justified by changes in chemotherapy protocols over the years, by the method of evaluating the incidence of OM in previous studies being raised per patient and not by evaluations as was done in this work and by way of treating and preventing these lesions nowadays, observing a reduction over time (13,19).

It is possible to note this change in the incidence of OM in our study, which shows a decrease in the prevalence of OM from the year 2018 to 2019. Furthermore, most published studies evaluating the incidence of MO have a study population under inpatient care, unlike our study, which evaluates patients under outpatient care (13,19-21).

Contrary to previous studies, such as that of Çakmak *et al*. (13), being female, having age between 45-55 years, and undergoing more than ten cycles of QT increased the risk of developing OM. However, in multivariate analysis, the independent risk factor for this development was the number of cycles, showing a very high risk in patients undergoing more than ten cycles of chemotherapy. These divergent findings are justified by the fact that the researchers performed the assessments of the patients when they were on at least their second round of chemotherapy. Other studies, such as that of Raber-Durlac *et al*. (19), showed in a multivariate analysis that chemotherapy cycles are not only involved with the risk of developing OM but also with the degree of severity.

In our study, head and neck, breast, sarcoma, and liver tumors were directly associated with OM. However, in multivariate analysis, the primary tumor location did not influence the risk of OM incidence, thus suggesting it is strongly associated with the treatment protocols. Unlike another study that suggests that the distribution of the primary tumor may influence the incidence of OM, this may be explained by the small size of their sample and by not evaluating the overall solid tumor landscape by including only colon and breast tumors in the study (19). However, further studies with a larger and more homogeneous sample that broadly explore solid tumors and their relationship with primary tumor distribution and chemotherapy under the development of OM are needed.

In multivariate analysis, M1 tumors had a 0.21- and 0.38-times lower risk of developing BM. Patients who have distant metastases and were undergoing palliative treatment usually encompass reduced doses of chemotherapy drugs to maintain quality of life, which may reduce the incidence of OM (22,23).

The chemotherapeutic agents that influenced the prevalence of oral mucositis were cyclophosphamide, docetaxel, doxorubicin, trastuzumab, and atezolizumab and in the multivariate analysis, only carboplatin, cisplatin, docetaxel, and bevacizumab significantly increased the prevalence of mucositis. A study conducted in 2010 in Austria also assessed similarly to this study the relationship of the incidence of OM in solid tumors, and it was observed that the main chemotherapeutic agents involved in the development of OM were taxanes cisplatin and irinotecan (24).

About 92% of the 1,262 patients with head and neck tumors had RT and used cisplatin-based chemotherapy (50.4%), followed by carboplatin (36.1%) and paclitaxel (32.3%). Most patients treated for head and neck cancer are commonly submitted to RT, which may be conventional or hyperfractionated (25). Studies show that the association of RT and chemotherapy increases the incidence, severity, and duration of oral mucositis, especially when combinations of different drugs and hyperfractionation schemes are used (25,26).

In this study, the findings are similar; patients who performed head and neck RT obtained a significantly higher prevalence of OM. This is also compatible with the data obtained in a systematic review, where it was observed that patients who underwent fractionated RT had an impact of severity and incidence of oral mucositis of 34%, whereas those who received CRT had this impact of 43% (27).

The use of CRT is common in tumors in advanced stages and with the presence of positive lymph nodes, which corroborates the findings of higher prevalence of BM in these cases (27). Moreover, patients who performed more than three cycles and had high age and BMI had a higher prevalence of BM, which is common for patients who perform more cycles of QT and have higher age to have greater toxicity, mainly due to changes in renal function and difficulty in excreting the drug (23).

Monotherapies showed a higher prevalence of mucositis than therapies involving two or more chemotherapies, noting different results from previous studies that point out that combination chemotherapies in conjunction with RT increase the incidence and severity of OM (25,26).

Despite the limitation of being a retrospective study and the difficulty in obtaining some information, especially if follow up, this study synthesizes essential information about risk factors for the treatment of solid tumors only, a population that has been little explored in studies of oral adverse effects.

Thus, we conclude that patients with solid tumors who undergo treatment with QT have a low prevalence of OM, but women, middle-aged patients (45-55 years), with high BMI, who undergo more than six cycles of chemotherapy and are treated with carboplatin, cisplatin, docetaxel, and bevacizumab are important risk groups. Additionally, in the head and neck region, RT, especially in combination with carboplatin, is the most critical risk factor for OM.

## References

[1] Rallis KS, Lai Yau TH, Sideris M (2021). Chemoradiotherapy in Cancer Treatment: Rationale and Clinical Applications. Anticancer Res.

[2] Chaveli-López B (2014). Oral toxicity produced by chemotherapy: A systematic review. J Clin Exp Dent.

[3] Gamper EM, Giesinger JM, Oberguggenberger A, Kemmler G, Wintner LM, Gattringer K (2012). Taste alterations in breast and gynaecological cancer patients receiving chemotherapy: prevalence, course of severity, and quality of life correlates. Acta Oncol.

[4] Selle F, Gligorov J, Soares DG, Lotz JP (2016). High-dose chemotherapy as a strategy to overcome drug resistance in solid tumors. Bull Cancer.

[5] Jones JA, Avritscher EB, Cooksley CD, Michelet M, Bekele BN, Elting LS (2020). Epidemiology of treatment associated mucosal injury after treatment with newer regimens for lymphoma, breast, lung, or colorectal cancer. Support Care Cancer.

[6] Elting LS, Cooksley C, Chambers M, Cantor SB, Manzullo E, Rubenstein EB (2003). The burdens of cancer therapy: clinical and economic outcomes of chemotherapy‐induced mucositis. Cancer.

[7] Kuba S, Fujiyama R, Yamanouchi K, Morita M, Sakimura C, Hatachi T (2018). Awareness of dysgeusia and gustatory tests in patients undergoing chemotherapy for breast cancer. Support Care Cancer.

[8] Sroussi HY, Epstein JB, Bensadoun RJ, Saunders DP, Lalla RV, Migliorati CA (2017). Common oral complications of head and neck cancer radiation therapy: mucositis, infections, saliva change, fibrosis, sensory dysfunctions, dental caries, periodontal disease, and osteoradionecrosis. Cancer Med.

[9] Lalla RV, Saunders DP, Peterson DE (2014). Chemotherapy or radiation-induced oral mucositis. Dent Clin N Am.

[10] Al-Dasooqi N, Sonis ST, Bowen JM, Bateman E, Blijlevens N, Gibson RJ (2013). Emerging evidence on the pathobiology of mucositis. Support Care Cancer.

[11] Freites-Martinez A, Santana N, Arias-Santiago S, Viera A (2021). Using the Common Terminology Criteria for Adverse Events (CTCAE - Version 5.0) to Evaluate the Severity of Adverse Events of Anticancer Therapies. Actas Dermosifiliogr (Engl Ed).

[12] Nagatani A, Ogawa Y, Sunaga T, Tomura K, Naito Y, Fujii N (2017). Analysis of the Risk Factors for Severe Oral Mucositis in Head and Neck Cancer after Chemoradiotherapy with S-1. Yakugaku Zasshi.

[13] Çakmak S, Nural N (2019). Incidence of and risk factors for development of oral mucositis in outpatients undergoing cancer chemotherapy. Int J Nurs Pract.

[14] Barasch A, Peterson DE (2003). Risk factors for ulcerative oral mucositis in cancer patients: unanswered questions. Oral Oncol.

[15] Münstedt K, Männle H (2019). Using bee products for the prevention and treatment of oral mucositis induced by cancer treatment. Molecules.

[16] Oberoi S, Zamperlini-Netto G, Beyene J, Treister NS, Sung L (2019). Effect of prophylactic low level laser therapy on oral mucositis: a systematic review and meta-analysis. PLoSone.

[17] Malta M, Cardoso LO, Bastos FI, Magnanini MMF, Silva CMFP (2010). STROBE initiative: guidelines on reporting observational studies. Rev Saúde Públ.

[18] Amin MB, Greene FL, Edge SB, Compton CC, Gershenwald JE, Brookland RK (2017). The Eighth Edition AJCC Cancer Staging Manual: Continuing to build a bridge from a population-based to a more "personalized" approach to cancer staging. CA Cancer J Clin.

[19] Raber-Durlacher JE, Weijl NI, Abu Saris M, de Koning B, Zwinderman AH, Osanto S (2001). Oral mucositis in patients treated with chemotherapy for solid tumors: a retrospective analysis of 150 cases. Support Care Cancer.

[20] Cheng KKF, Leung SF, Liang RH, Tai JW, Yeung RM, Thompfilho DR (2010). Severe oral mucositis associated with cancer treatment: impact on oral function and quality of life. Support Care Cancer.

[21] Al-Rudayni AHM, Gopinath D, Maharajan MK, Menon RK (2020). Impact of oral mucositis on quality of life in patients undergoing oncological treatment: a systematic review. Transl Cancer Res.

[22] Haddad RI, Posner M, Hitt R, Cohen EW, Schulten J, Lefebvre JL (2018). Induction chemotherapy in locally advanced squamous cell carcinoma of the head and neck: role, controversy, and future directions. Ann Oncol.

[23] Porock D, Nikoletti S, Cameron F (2004). The relationship between factors that impair wound healing and the severity of acute radiation skin and mucosal toxicities in head and neck cancer. Cancer Nurs.

[24] Wuketich S, Hienz SA, Marosi C (2011). Prevalence of clinically relevant oral mucositis in outpatients receiving myelosuppressive chemotherapy for solid tumors. Support Care Cancer.

[25] Duncan GG, Epstein JB, Tu D, El Sayed S, Bezjak A, Ottaway J (2005). Quality of life, mucositis, and xerostomia from radiotherapy for head and neck cancers: a report from the NCIC CTG HN2 randomized trial of an antimicrobial lozenge to prevent mucositis. Head Neck.

[26] Ang KK, Zhang Q, Rosenthal DI, Nguyen-Tan PF, Sherman EJ, Weber RS (2014). Randomized phase III trial of concurrent accelerated radiation plus cisplatin with or without cetuximab for stage III to IV head and neck carcinoma: RTOG 0522. J Clin Oncol.

[27] Trotti A, Bellm LA, Epstein JB, Frame D, Fuchs HJ, Gwede CK (2003). Mucositis incidence, severity and associated outcomes in patients with head and neck cancer receiving radiotherapy with or without chemotherapy: a systematic literature review. Radiother Oncol.

